# Bacterial Signaling Nucleotides Inhibit Yeast Cell Growth by Impacting Mitochondrial and Other Specifically Eukaryotic Functions

**DOI:** 10.1128/mBio.01047-17

**Published:** 2017-07-25

**Authors:** Andy Hesketh, Marta Vergnano, Chris Wan, Stephen G. Oliver

**Affiliations:** a Cambridge Systems Biology Centre, University of Cambridge, Cambridge, United Kingdom; b Department of Biochemistry, University of Cambridge, Cambridge, United Kingdom; University of British Columbia

**Keywords:** *Saccharomyces cerevisiae*, cyclic nucleotides, host-pathogen interactions, signaling nucleotides, yeasts

## Abstract

We have engineered *Saccharomyces cerevisiae* to inducibly synthesize the prokaryotic signaling nucleotides cyclic di-GMP (cdiGMP), cdiAMP, and ppGpp in order to characterize the range of effects these nucleotides exert on eukaryotic cell function during bacterial pathogenesis. Synthetic genetic array (SGA) and transcriptome analyses indicated that, while these compounds elicit some common reactions in yeast, there are also complex and distinctive responses to each of the three nucleotides. All three are capable of inhibiting eukaryotic cell growth, with the guanine nucleotides exhibiting stronger effects than cdiAMP. Mutations compromising mitochondrial function and chromatin remodeling show negative epistatic interactions with all three nucleotides. In contrast, certain mutations that cause defects in chromatin modification and ribosomal protein function show positive epistasis, alleviating growth inhibition by at least two of the three nucleotides. Uniquely, cdiGMP is lethal both to cells growing by respiration on acetate and to obligately fermentative petite mutants. cdiGMP is also synthetically lethal with the ribonucleotide reductase (RNR) inhibitor hydroxyurea. Heterologous expression of the human ppGpp hydrolase Mesh1p prevented the accumulation of ppGpp in the engineered yeast and restored cell growth. Extensive *in vivo* interactions between bacterial signaling molecules and eukaryotic gene function occur, resulting in outcomes ranging from growth inhibition to death. cdiGMP functions through a mechanism that must be compensated by unhindered RNR activity or by functionally competent mitochondria. Mesh1p may be required for abrogating the damaging effects of ppGpp in human cells subjected to bacterial infection.

## IMPORTANCE

During infections, pathogenic bacteria can release nucleotides into the cells of their eukaryotic hosts. These nucleotides are recognized as signals that contribute to the initiation of defensive immune responses that help the infected cells recover. Despite the importance of this process, the broader impact of bacterial nucleotides on the functioning of eukaryotic cells remains poorly defined. To address this, we genetically modified cells of the eukaryote *Saccharomyces cerevisiae* (baker’s yeast) to produce three of these molecules (cdiAMP, cdiGMP, and ppGpp) and used the engineered strains as model systems to characterize the effects of the molecules on the cells. In addition to demonstrating that the nucleotides are each capable of adversely affecting yeast cell function and growth, we also identified the cellular functions important for mitigating the damage caused, suggesting possible modes of action. This study expands our understanding of the molecular interactions that can take place between bacterial and eukaryotic cells.

## INTRODUCTION

Many bacteria rely on the synthesis of nucleotide signaling molecules to effect changes in their metabolism and to coordinate survival programs that help them persist through periods of perceived stress (1–4). In species that are plant or animal pathogens, these signaling networks are often extended to contribute to virulence and survival in the host. The highly phosphorylated linear guanosine alarmone, ppGpp, is synthesized in response to nutrient starvation signals and is an important factor in the establishment and maintenance of infections caused by a wide range of Gram-negative and Gram-positive pathogens (5, 6). The related cyclic dinucleotide monophosphate molecules cyclic di-AMP (cdiAMP) and cdiGMP also help coordinate physiological responses, including biofilm formation, motility, and stress resistance, which can influence pathogenesis in diverse bacterial species (7–13). Interestingly, the presence of these cyclic dinucleotides in animal cells can be sensed by STING (stimulator of interferon genes) to activate the innate immune response (14–16) and can also mediate a STING-independent activation of the NLRP3 inflammasome (17). cdiGMP and cdiAMP are therefore significant participants in the metabolic cross talk between host and pathogen, as they are required both for the effective functioning of the invading bacterial cells and for their detection by the host’s defenses. Apart from their induction of host immune responses, other influences of these bacterial signaling molecules on host cell physiology and metabolism remain poorly defined. Unique among higher eukaryotes, plant cells express ppGpp synthetase activity in plastids and produce ppGpp in chloroplasts in response to both environmental and wounding stresses (18, 19). Heterologous expression of a plant ppGpp synthetase in yeast cells was found to improve cell survival under a range of stress conditions, and preliminary microarray data (lacking replication) suggested effects on yeast gene transcription (20). Thus, a study of the impact of these bacterial nucleotides on eukaryotic cell function has great relevance to the interaction of bacteria with their hosts, including both pathogenic and symbiotic relationships with plants and animals (21–23).

To determine the effects of the presence of ppGpp, cdiAMP, and cdiGMP in the cytosol on the functioning of a eukaryotic cell, we heterologously expressed enzymes for their synthesis in the budding yeast *Saccharomyces cerevisiae*. Synthetic genetic array (SGA) analysis (24) was used to help characterize the cellular processes most affected by the synthesis of each molecule, and transcriptome analysis was employed to measure any effects on gene transcription. The results defined a significant activity for the guanine nucleotides related to respiration and mitochondrial function. The impact of intracellular cdiAMP on yeast cells was less marked, and the expression levels of only 75 genes were significantly altered following intracellular accumulation of cdiAMP to levels >5-fold higher than those observed for ATP.

## RESULTS

### Inducible synthesis of bacterial nucleotides in *S. cerevisiae.*

**FIG 1:**
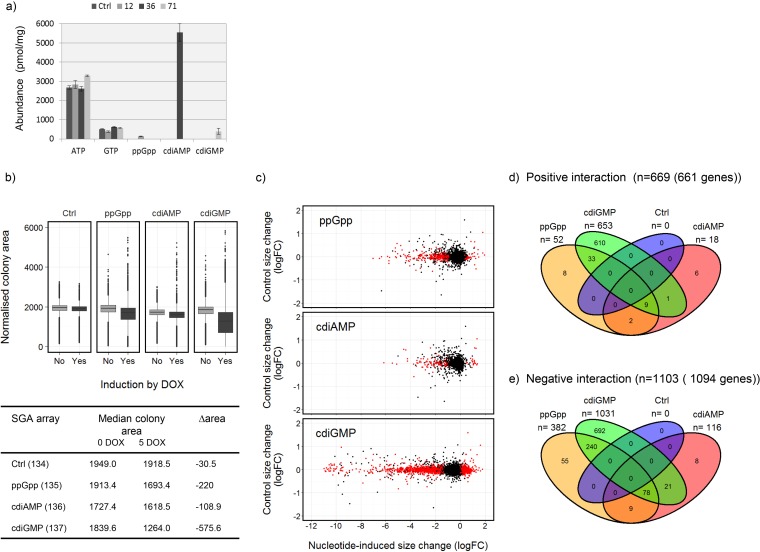
Yeast gene mutations exhibiting interactions with synthesis of the bacterial nucleotides ppGpp, cdiAMP, and cdiGMP (Data Set S1). (a) DOX-inducible synthesis of bacterial nucleotides in yeast cells. Strains CML282-252 (Ctrl), CML282-12 (ppGpp), CML282-36 (cdiAMP), and CML282-71 (cdiGMP) were induced with DOX (10 μg/ml), and intracellular nucleotide composition was analyzed by LC-MS. Nucleotide abundances (normalized to picomoles per milligram [dry weight] of cells) are the averages of duplicate cultures (± standard deviation): ppGpp, 131 (±6.5); cdiAMP 5,553 (±456); cdiGMP 397 (±164). (b) SGA analysis showing changes in normalized colony size distribution following DOX induction of nucleotide synthesis (4,990 strains spotted in replicates of 4): 134, control array derived from Y7092-112-134; 135, ppGpp array derived from Y7092-112-135; 136, cdiAMP array derived from Y7092-112-136; 137, cdiGMP array derived from Y7092-112-137. (c) Changes in the median normalized colony size (*n* = 4) of each mutant strain in the control SGA upon DOX induction versus the changes observed in the corresponding arrays synthesizing ppGpp, cdiAMP, or cdiGMP. Mutants highlighted in red exhibited interactions, identified from the mutants showing no interaction in the control query strain pAH134 (0.7 ≥ *Wij* pAH134 ≤ 1.3; *n* = 4,916), showing colony size changes that were significantly different (*q* < 0.05) from the expected after analysis using the Treat function in Limma and with calculated *S* interaction scores of >0.3 (positive interaction) or less than −0.3 (negative interaction). (d and e) Venn diagrams showing the mutations identified in panel c that interacted positively (d) or negatively (e) with each nucleotide.

To generate strains of *S. cerevisiae* where the intracellular synthesis of ppGpp, cdiGMP, and cdiAMP nucleotides could be controllably induced, we heterologously expressed genes (or portions of genes) encoding selected bacterial enzymes specifying their synthesis by using the dual tetracycline activator/repressor system of Belli et al. (25). A *Caulobacter crescentus* gene variant, *dgcA0244*, which encodes a synthetase that is insensitive to product feedback inhibition, was chosen to provide cdiGMP synthesis (26), while genes generating truncated enzymes which exhibit constitutively active synthetase activity were used for cdiAMP and ppGpp production. These genes were based on *ybbP* from *Bacillus subtilis* (27) and *relA* from *Escherichia coli* (28), respectively. In addition to the Tet-ON-driven expression of the target gene, which can be induced by addition of doxycycline (DOX), the dual-tetracycline activator/repressor system also includes an SSN6::TetR repressor construct integrated into the chromosome which enables the complete switching off of the regulated gene in the absence of inducer. The functionalities of the inducible synthetase constructs were initially tested using the BMA64-1A derivative CML282 (25, 29) as the host strain and analyzing the intracellular nucleotide composition following induction with DOX during growth in batch culture (Fig. 1a). Induction of the *relA* gene fragment via DOX yielded ca. 130 pmol of ppGpp/mg (dry weight) of cells in strain CML282-12, compared to ca. 400 and 5,500 pmol/mg for cdiGMP and cdiAMP, respectively, in strains CML282-71 and CML282-36 (Fig. 1a). The synthesis systems were then also constructed in the BY4741 genetic background to permit directly comparable results of transcriptome and SGA analyses (see below). All BY4741 derivatives were constructed to produce prototrophic strains in order to eliminate confounding effects that could arise from the metabolic consequences of auxotrophy (30). The level of production of all three bacterial nucleotides showed some dependence on the strain background (e.g., compare Fig. 1a with Table 1), but the order of effectiveness of the expression systems was conserved: cdiAMP > cdiGMP > ppGpp. In both backgrounds, bacterial nucleotide synthesis was undetectable in either the control strain or the expression strains in the absence of inducer.

10.1128/mBio.01047-17.4 DATA SET S1  SGA analysis results that identified gene deletions that positively or negatively interacted with the synthesis of a particular nucleotide. Download DATA SET S1, XLSX file, 7.6 MB. Copyright © 2017 Hesketh et al. 2017 Hesketh et al. This content is distributed under the terms of the Creative Commons Attribution 4.0 International license.

**TABLE 1:** Summary of yeast genes whose transcription was significantly (q < 0.05) affected following induction of at least one of the bacterial nucleotides in exponentially growing cultures

Strain	Nucleotide abundance^a^	No. of upregulated genes	No. of downregulated genes
BY4741-112-134		0	0
BY4741-112-135	ppGpp (102 ± 20)	80	28
BY4741-112-136	cdiAMP (9,978 ± 1,014)	58	17
BY4741-112-137	cdiGMP (3,273± 307)	17	49

a Abundance is reported as the picomoles of the nucleotide per milligram (dry weight) of cells. Means ± standard deviations are shown in parentheses.

### ppGpp, cdiAMP, and cdiGMP affect yeast cell function.

To assess the activity of bacterial nucleotides on yeast cell function and to identify cellular processes that may buffer the pathway(s) targeted by them, gene mutations which interact synergistically with DOX-dependent induction of their synthesis were sought by using SGA analysis. In classical SGA analysis, as developed by Boone and his collaborators (31), functional interactions between genes are revealed by crossing a strain with a deletion for a query gene with all viable single-gene deletion mutants of yeast. The doubly heterozygous strains are then allowed to sporulate and the haploid double mutants are selected, and their phenotypes are compared with those of the two parent strains. In our SGA analysis, the interaction between the production of the bacterial nucleotides and each viable single-gene yeast deletion mutant was assessed in order to reveal genes that represented targets for these nucleotides, or which aggravated or ameliorated their impact on yeast. Thus, each of the vectors conferring bacterial nucleotide synthesis was incorporated into the mutant library of haploid yeasts, each of which had a deleted nonessential gene, by using the SGA methodology (24, 32) (see Materials and Methods). Transformation into a derivative of the SGA query strain Y7092 (24), which carries an integrated copy of the SSN6::TetR repressor obtained from pCM242 (25), generated a collection of strains that did not mediate bacterial nucleotide synthesis in the absence of DOX and which possessed a full complement of wild-type genes to complement the auxotrophic markers in the background strain and thus enabled prototrophic growth.

The output arrays were screened on a defined minimal medium to identify interactions between gene mutations and the induction of the nucleotide synthesis constructs, thereby revealing any changes in the functional organization of yeast cells containing the bacterial signaling molecules (Fig. 1; see also Data Set S1 in the supplemental material). All three nucleotide synthesis arrays showed a generalized decrease in fitness following induction with DOX, with the guanine nucleotides cdiGMP and ppGpp causing the largest effects (Fig. 1b and c). For each synthesis strain, this decrease in fitness (given by the ratio of the median colony size on DOX plates to the median size on plates lacking DOX) was taken as the expected effect of induction and used as a baseline from which to look for mutations that synthetically alleviated (a positive interaction) or aggravated (a negative interaction) the expected fitness defect (FD) (Fig. 1c to e). No significant interactions were identified for the control strain, while the numbers for the nucleotide synthesis strains increased in the order of cdiGMP > ppGpp > cdiAMP (Fig. 1e). Many genes showed interactions with more than one nucleotide.

### Mutations compromising mitochondrial function and chromatin remodeling show synthetic sick interactions with the synthesis of ppGpp, cdiAMP, and cdiGMP.

**FIG 2:**
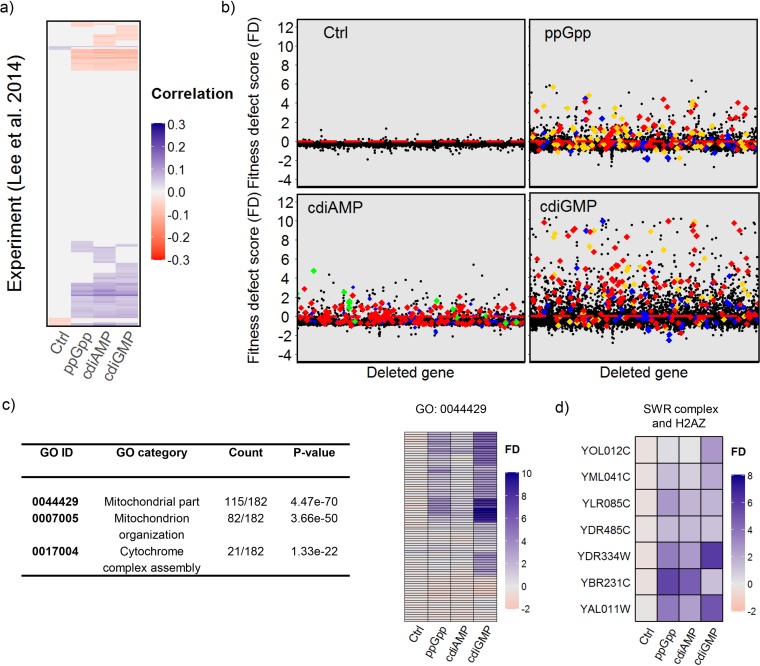
ppGpp, cdiAMP, and cdiGMP reduce the fitness of yeast mutants with compromised SWR1 complex activity or mitochondrial function. (a) Pearson correlation of FD scores with data from the chemogenomic experiments of Lee et al. (33). Only significant correlations are shown (*q ≤* 0.01). (b) Change in colony size induced by the synthesis of each nucleotide in each mutant. Colored points correspond to selected chemogenomic profile signature groups of Lee et al. (33) and indicate the highest positive correlations with the SGA fitness profiles observed for the bacterial nucleotides. Gold, mitochondrial stress; green, tubulin folding and SWR complex; red, minor response 78; blue, translation. (c) Gene ontology analysis of the genes in the minor response 78 signature showed significant enrichment for mitochondrial functions. The heat map shows FD scores for the 115 genes from this signature which were in the mitochondrial portion of the GO category (0044429), indicating marked fitness defects, particularly in the presence of ppGpp and cdiGMP. (d) Heat map of FD scores for 6 genes belonging to the SWR1 complex that showed negative, synthetic sick interactions with the synthesis of each nucleotide. The negative interaction with *HTZ1* (YOL012C) encoding the H2AZ histone variant exchanged by the SWR1 complex is also shown.

Chemical genomics studies in *S. cerevisiae* have been used previously to assess the influence of a library of 3,250 small molecules on survival of homozygous diploid mutant strains in pooled growth competition assays (33). In this way, the *in vivo* mechanism of action of each drug tested was classified into a response signature according to its effect on mutant survival. To help identify cellular processes significantly affected by ppGpp, cdiAMP, and cdiGMP, correlations between the SGA interactions identified for the nucleotide synthesis strains and the published yeast chemogenomic data were calculated (Fig. 2; Data Set S2). Weak but significant (*q* ≤ 0.01) correlations with ca. 20% of the experiments based on the chemogenomics data were identified for each nucleotide, while the control strain exhibited far fewer (Fig. 2a). The data for ppGpp and cdiGMP correlated most highly with experimental results that Lee et al. (33) assigned to the “mitochondrial stress” signature and also to the unknown minor response signature 78, which is significantly enriched for functions associated with respiration and mitochondrial organization (Fig. 2b and c; Data Set S3). cdiAMP produced less frequent fitness defects and these were of lower magnitude and correlated with signatures assigned to “tubulin folding and SWR complex” and “translation,” in addition to “mitochondrial stress” and minor response signature 78 (Fig. 2; Data Set S2).

10.1128/mBio.01047-17.5 DATA SET S2  Correlations between the fitness changes observed in the nucleotide synthesis SGA screens and published fitness defect (FD) scores from chemogenomic profiling analysis of homozygous diploid mutants (Lee et al. 2014 [33]). Download DATA SET S2, XLSX file, 1.2 MB. Copyright © 2017 Hesketh et al. 2017 Hesketh et al. This content is distributed under the terms of the Creative Commons Attribution 4.0 International license.

10.1128/mBio.01047-17.6 DATA SET S3  GO analysis of the groups of genes identified as interacting positively or negatively with ppGpp, cdiAMP, or cdiGMP (as illustrated in Fig. 1d). Download DATA SET S3, XLSX file, 0.4 MB. Copyright © 2017 Hesketh et al. 2017 Hesketh et al. This content is distributed under the terms of the Creative Commons Attribution 4.0 International license.

Closer inspection of the FD scores for the gene deletions in the tubulin folding and SWR complex response indicated that six genes from the SWR complex, plus *HTZ1* (YOL012C), which encodes the SWR1 complex substrate histone H2AZ, synergistically reduced fitness with each of the bacterial nucleotides (Fig. 2d). The SWR complex gene ontology (GO) category 0000812 was also significantly enriched in the groups of genes showing negative interactions with the synthesis of each nucleotide (ppGpp, *P* = 1.45e−5; cdiAMP, *P* = 9.62e−9; cdiGMP, *P* = 4.09e−4) (Data Set S3; Fig. S1). GO analysis similarly identified mitotic sister chromatid cohesion (0007064) as an enriched category in all the negative interaction groups (ppGpp, *P* = 8.07e−6; cdiAMP, *P* = 2.06e−4; cdiGMP, *P* = 5.49e−4), and several related categories associated with chromatin remodeling in the negative interactions for both ppGpp and cdiAMP (Data Set S3, Fig. S1). Negative interactions with ppGpp were additionally enriched for the cell cycle and kinetochore GO categories, while the Golgi subcompartment was identified for both cdiAMP and cdiGMP (Data Set S3, Fig. S1).

10.1128/mBio.01047-17.1 FIG S1  Network of GO categories significantly (*P* ≤ 0.001) enriched in the groups of genes that showed synthetic negative interactions with each nucleotide (based on GO analysis results presented in Data Set S1). Download FIG S1, PDF file, 0.5 MB. Copyright © 2017 Hesketh et al. 2017 Hesketh et al. This content is distributed under the terms of the Creative Commons Attribution 4.0 International license.

### cdiGMP is synthetically lethal with both the petite mutation and under exclusively respiratory metabolic conditions.

**FIG 3:**
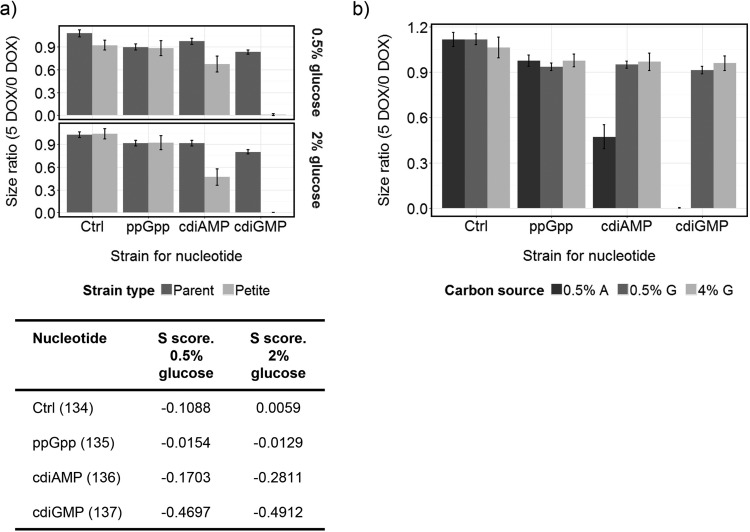
(a and b) cdiGMP is lethal in both petite mutant cells growing fermentatively (a) and during exclusively respiratory growth on acetate (b). (a) The petite mutant derivatives of BY4741-112-134 (Ctrl), BY4741-112-135 (ppGpp), BY4741-112-136 (cdiAMP), and BY4741-112-137 (cdiGMP) were generated and analyzed to determine the effect of inducing pathway expression on colony size. Results, expressed as the ratio of the induced colony areas versus those of noninduced controls, are shown in comparison to the effects on parental nonpetite (parent) strains. Two different glucose concentrations were used in the media: 0.5% or 2%. The data for the petites are from the analysis of four independently isolated mutants, spotted 8 times each. The nonpetite strains were spotted 24 times each. Mean size changes (± standard errors; *n* = 32 for petites, *n* = 24 for parents) are shown alongside interaction (*S*) scores and were calculated based on the changes in colony areas. Strongly negative values (less than −0.1) indicate synthetic sick interactions between petiteness and nucleotide synthesis. (b) The parental strains were arrayed onto YNB minimal medium plates containing 0.5% acetate (0.5% A), 0.5% glucose (0.5% G), or 4% glucose (4% G), with (5 DOX) or without (0 DOX) doxycycline. Strains were spotted 24 times each, and the data were collected and analyzed as described for panel a. The plates containing glucose or acetate were scanned after 3 days of growth.

To further investigate the suggested relationship between mitochondrial function and bacterial nucleotide synthesis, petite mutants lacking mitochondrial DNA were generated from a series of strains in the BY4741-112 background by treatment with ethidium bromide. Petite mutants cannot respire and can only grow via fermentative metabolism. Strikingly, cdiGMP synthesis was lethal in the petite background during growth on yeast nitrogen base (YNB)-glucose minimal medium, while ppGpp showed no interaction with the mutation (Fig. 3a). cdiAMP exhibited an intermediate (synthetic sick) phenotype. To contrast these results, conditions under which yeast cells rely exclusively on mitochondrial respiration can be created using growth on a nonfermentative carbon source, such as acetate. Interestingly, induction of nucleotide synthesis under these conditions produced a similar pattern of effects with the petite mutation (Fig. 3b). Thus, cdiGMP is lethal, cdiAMP inhibits growth on acetate more than growth on glucose, and ppGpp exhibits similar inhibitory effects on either carbon source.

### Gene deletions that positively interact with nucleotide synthesis are associated with translation and chromatin dynamics.

**FIG 4:**
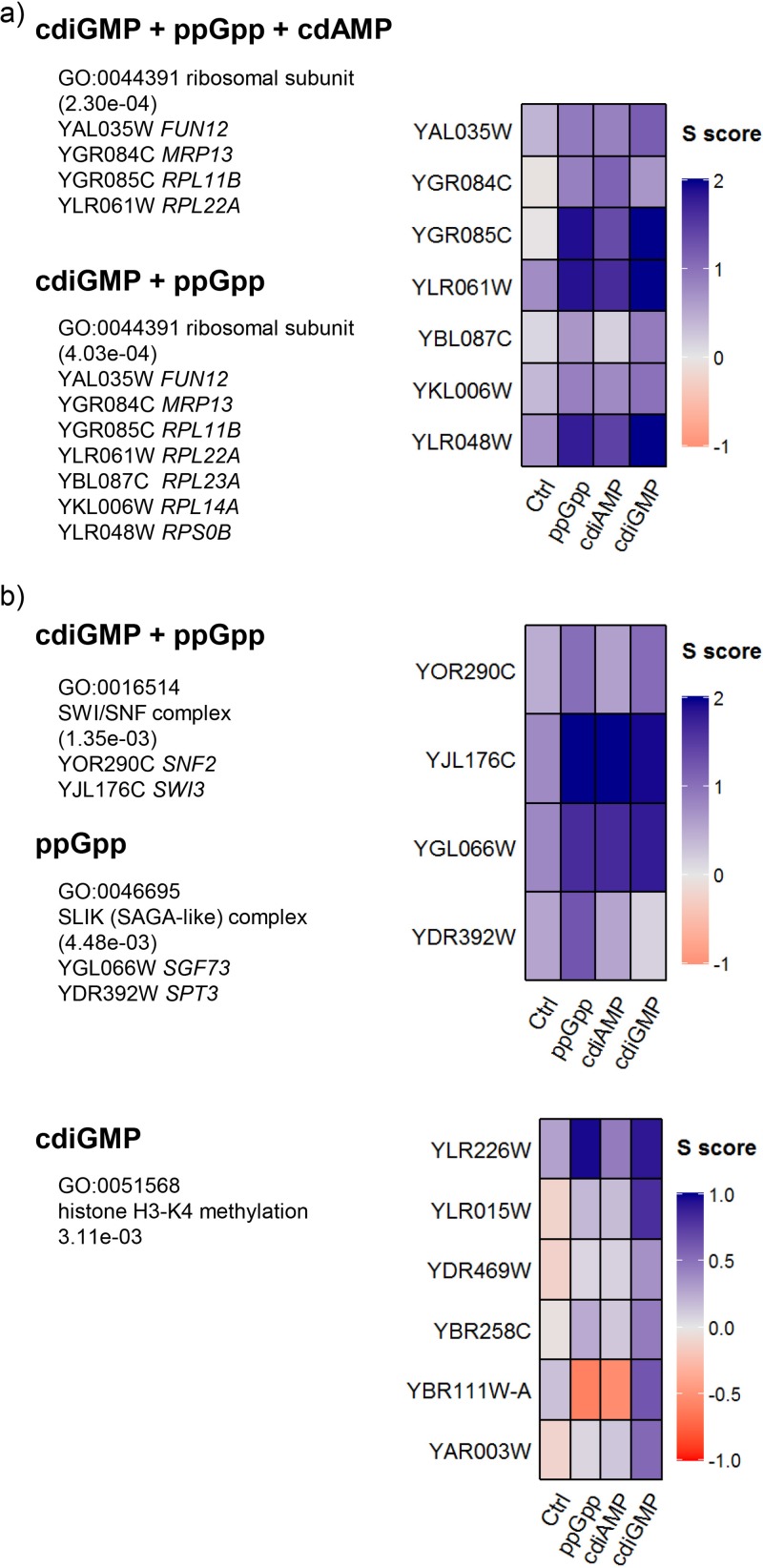
Analysis of the genes identified as positively interacting with the synthesis of the bacterial nucleotides identified the translational machinery (a) and chromatin modifications (b) as important functions. A selected summary of the GO analysis results from Data Set S3 are presented, accompanied by heat maps showing interaction SGA (*S*) scores for the genes in the categories of interest. In each case, the enrichments illustrated correspond to the positively interacting genes shared between (or limited to) the nucleotides listed.

The nine genes identified as positively interacting with the synthesis of all the nucleotides showed enrichment for the ribosomal subunit GO category 0044391 (*P* = 2.30e−4) (Fig. 4; Data Set S3). This includes three genes that encode subunits of the cytosolic ribosome (*RPL11B*, *RPL22A*, and *FUN12*) and one (*MRP13*) that specifyies a mitochondrial ribosomal protein. *RPL11B* and *MRP13* are adjacent on chromosome VII and may represent a single positive epistasis locus. Interestingly, two of the remaining genes in this small group (*SWI3* and *SGF73*) encode subunits of chromatin modification complexes (Fig. 4). Positive interactions shared between ppGpp and cdiGMP additionally identified *SNF2*, which encodes the catalytic subunit of the SWI/SNF chromatin remodeling complex, and three genes specifying cytoribosomal subunits (*RPL14A*, *RPL23A*, and *RPS0B*) (Fig. 4). The SWI/SNF complex GO category 0016514 (*P* = 1.35e−3) is also enriched for genes showing positive epistasis with both ppGpp and cdiGMP. Many more genes that positively interact with cdiGMP synthesis were identified than for those that positively interact either cdiAMP or ppGpp (Data Set S5), perhaps reflecting the stronger growth inhibitory effect of this nucleotide (Fig. 1). GO analysis of this group of genes identified enrichment for histone H3-K4 methylation (0051568; *P* = 3.11e−3) category, providing additional support for the significance of chromatin dynamics in the response to this nucleotide (Fig. 4; Data Set S3).

### ppGpp upregulates transcription of genes involved in energy balance and production.

Transcriptome analysis identified 127 genes whose expression was significantly upregulated at the 5% probability level upon inducing the synthesis of at least one of the bacterial nucleotides in exponentially growing yeast cells, and 65 genes were downregulated (Table 1; Fig. S2 and Data Set S4). Consistent with previous results, no significant changes in gene expression were identified as a result of DOX addition to the control strain (34). Synthesis of ppGpp elicited the largest number of significant changes in transcription, even though it accumulated to markedly lower levels inside the cells.

10.1128/mBio.01047-17.2 FIG S2  Changes in yeast gene transcription levels caused by the bacterial nucleotides ppGpp, cdiAMP, and cdiGMP. (a) Liquid cultures treated (+) or not (−) with 5 μg/ml DOX were grown to mid-exponential phase and analyzed for bacterial nucleotide synthesis via HPLC-UV. (b and c) RNA was isolated from aliquots of the cultures, and mRNA abundance levels were quantified using Affymetrix microarrays (a) and PCA analysis (b), which indicated changes in the pattern of gene transcription in the presence of each bacterial nucleotide (identified by differential expression testing via Limma treatment [*q* ≤ 0.05; 10% fold change threshold). (c) Venn diagrams summarizing genes identified as being significantly up- or downregulated by each nucleotide. Download FIG S2, PDF file, 0.2 MB. Copyright © 2017 Hesketh et al. 2017 Hesketh et al. This content is distributed under the terms of the Creative Commons Attribution 4.0 International license.

10.1128/mBio.01047-17.7 DATA SET S4  Transcriptomics analysis of the effect of inducing ppGpp, cdiAMP, or cdGMP synthesis in yeast cells. Data from hybridizations to Affymetrix Yeast 2.0 microarrays were RMA normalized, filtered, and analyzed for differential expression using the Treat function of Limma. Download DATA SET S4, XLSX file, 5.3 MB. Copyright © 2017 Hesketh et al. 2017 Hesketh et al. This content is distributed under the terms of the Creative Commons Attribution 4.0 International license.

**FIG 5:**
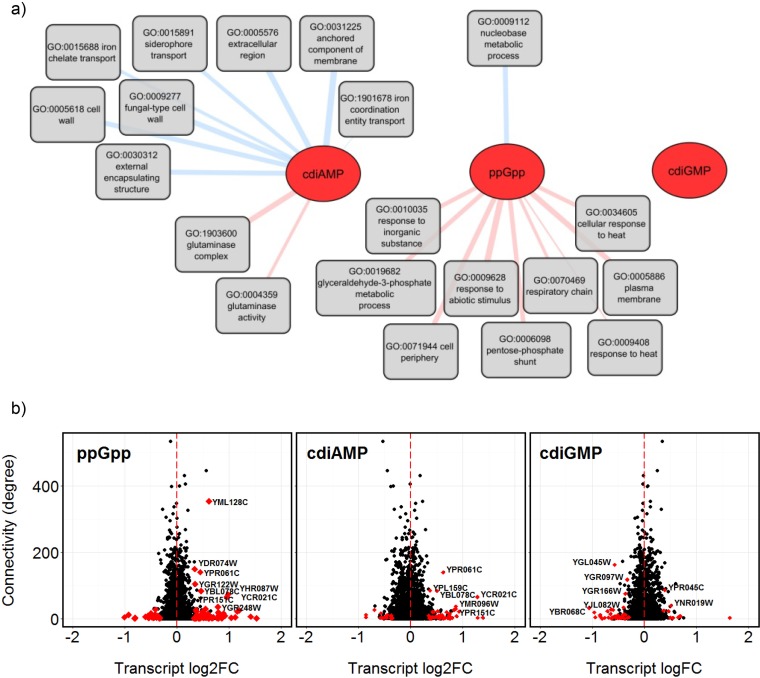
ppGpp upregulates transcription of genes involved in respiration, while cdiAMP exerts a response associated with cell envelope functions. (a) Network of GO categories significantly (*P* ≤ 0.001) enriched in the groups of genes upregulated (pink line) or downregulated (blue line) with each nucleotide (based on GO analysis results presented in Data Set S5). The line thickness is proportional to the *P* value, with thicker lines indicating greater significance. (b) Identification of genes highly connected in the *S. cerevisiae* stringent genetic interaction network that exhibited significant changes in transcription in response to ppGpp, cdiAMP, or cdiGMP based on the log_2_ fold change (FC) in transcript abundance, which was plotted against the degree of connectivity in the network. Red points indicate genes that were significantly differentially expressed, while names correspond to those that were differentially transcribed with a degree connectivity of ≥30.

GO analysis identified the “respiratory chain” category (0070469) (*P* = 5.43e−4) as being enriched in the group of genes significantly upregulated by ppGpp (Fig. 5a), including two genes YLR164W (*SHH4*) and YMR118C (*SHH3*), which encode paralogues of primary succinate dehydrogenase complex subunits, and two genes that encode cytochrome subunits expressed under hypoxic conditions [YEL039C (*CYC7*) and YIL111W (*COX5B*)] (Data Set S5). The implication that ppGpp causes an upregulation of specialized bioenergetic pathways is supported by the identification of the pentose phosphate shunt GO category (0006098; *P* = 3.35e−4) from the ppGpp-induced genes (Fig. 5a). This pathway allows the interconversion of C_3_, C_5_, and C_6_ sugars and functions to help balance the relative demands for ribose-5-phosphate, NADPH, and ATP, depending on metabolic status (35). Significant GO category enrichment was not observed among the 61 genes whose expression changed in response to cdiGMP, while those associated with cdiAMP whose expression level changed are listed in Fig. 5a and Data Set S5.

10.1128/mBio.01047-17.8 DATA SET S5  GO analysis of the groups of genes identified as being differentially expressed in yeast cells following induction of ppGpp, cdiAMP, or cdiGMP synthesis. Download DATA SET S5, XLSX file, 0.03 MB. Copyright © 2017 Hesketh et al. 2017 Hesketh et al. This content is distributed under the terms of the Creative Commons Attribution 4.0 International license.

The functional interconnectedness of the genes identified as being differently transcribed in the presence of each nucleotide was assessed using data from the stringent genetic interaction network for *S. cerevisiae* (36) (Fig. 5b; Data Set S6). A significant alteration in transcription of a more highly connected gene could be expected to result in broader changes in cell function. Genes upregulated by ppGpp or cdiAMP included several that are highly connected (degree, ≤30) in the stringent genetic interaction network, with four present in both sets: YPR151C (*SUE1*), YCR021C (*HSP30*), YBL078C (*ATG8*), and YPR061C (*JID1*). *ATG8*, *JID1*, and *SUE1* have roles in protein folding or degradation, with *SUE1* being required specifically for degradation of unstable forms of cytochrome *c* in mitochondria (37). Gene products from other highly connected genes upregulated by cdiAMP or ppGpp for which there is evidence supporting a mitochondrial function include YPL159C (*PET20*) and YML128C (*MSC1*). YGL045W (*RIM8*), which encodes a protein involved in proteolytic activation of Rim101p, was downregulated in response to cdiGMP.

10.1128/mBio.01047-17.9 DATA SET S6  Connectivity of the genes identified as being differently transcribed in the presence of each nucleotide in the stringent genetic interaction network for *S. cerevisiae* from Costanza et al. (36). Download DATA SET S6, XLSX file, 0.04 MB. Copyright © 2017 Hesketh et al. 2017 Hesketh et al. This content is distributed under the terms of the Creative Commons Attribution 4.0 International license.

### Ribonucleotide reductase activity is important for surviving exposure to cdiGMP.

**FIG 6:**
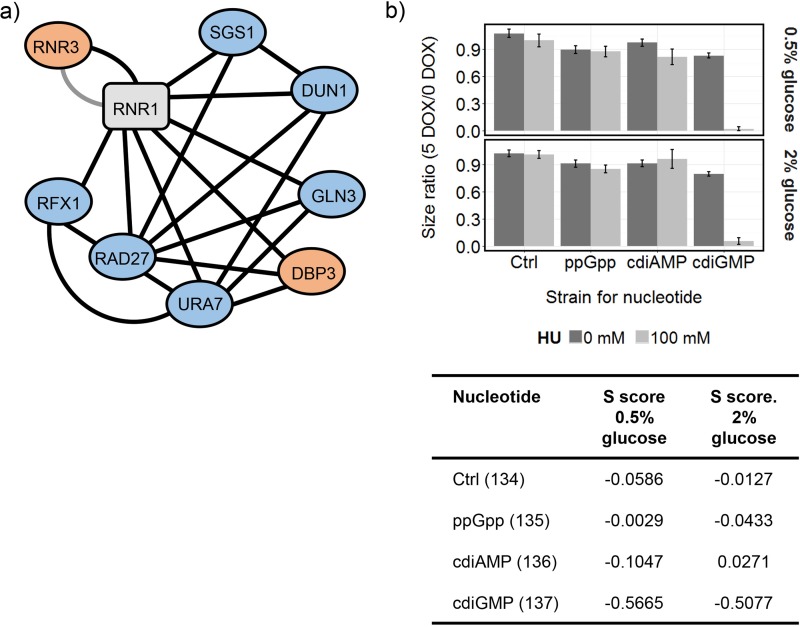
Ribonucleotide reductase activity is important for surviving exposure to cdiGMP. (a) Genes known to interact with RNR1 which synthetically alleviated (orange nodes) or aggravated (blue nodes) the effect of cdiGMP on cell fitness in the SGA screens. Edges connecting nodes are shown in black (genetic interaction) or gray (physical interaction) and are based on interaction data for RNR1 collated at http://www.yeastgenome.org. (b) BY4741-112-134 (Ctrl), BY4741-112-135 (ppGpp), BY4741-112-136 (cdiAMP), and BY4741-112-137 (cdiGMP) were arrayed onto YNB minimal medium plates containing 0 or 100 mM HU, with (5 DOX) or without (0 DOX) doxycycline. Strains were spotted 24 times each, and the data were collected and analyzed after 3 days of growth. Mean size changes (± standard errors; *n* = 24) are shown alongside interaction (*S*) scores, calculated based on the changes in colony areas. Strongly negative values (less than −0.1) indicate synthetic sick interactions between HU and nucleotide synthesis.

Among the 61 genes whose expression was significantly changed following induction of cdiGMP biosynthesis, the two most strongly upregulated, *HUG1* (7.4-fold) and *RNR3* (3.1-fold), were only induced in response to cdiGMP (Data Set S4), and both are known to influence ribonucleotide reductase (RNR) activity. Hug1p inhibits RNR activity via interaction with the Rnr2p subunit (38), while *RNR3* encodes a minor, nonessential isoform of the large RNR subunit. In addition, the SGA screens identified eight genes known to interact with *RNR1*, which encodes the major isoform of the large subunit of RNR, and this synthetically alleviated or aggravated the effect of cdiGMP (Fig. 6a). To test the hypothesis that RNR activity is important for coping with the effects of cdiGMP, bacterial nucleotide synthesis was induced in the presence of the RNR inhibitor hydroxyurea (HU) (Fig. 6). HU exhibited synthetic lethality with cdiGMP but showed no interaction with either ppGpp or cdiAMP synthesis (Fig. 6b).

### Rim101p and three homologous Mig transcription factors are potentially involved in adaptation to the bacterial nucleotides.

To identify transcription factors potentially involved in adaptation to the bacterial nucleotides, the promoter sequences upstream of the genes identified as being significantly differently expressed in response to each nucleotide were analyzed for enrichment with transcription factor binding sites by using data from the YEASTRACT repository of regulatory associations in *S. cerevisiae* (39) (Data Set S7). Transcription factor target sites identified as being significantly enriched in the groups of up- or downregulated genes suggest a role for the transcription factor in responding to the presence of the bacterial nucleotides. Genes upregulated in response to ppGpp and cdiAMP were enriched for regulatory associations with the stress-responsive transcription factors Msn2p and Msn4p and the pleiotropic drug resistance activators Pdr1p and Pdr3p, suggesting activation of these systems in response to each nucleotide. Individual deletion of the genes encoding these transcription factors had no detrimental effect on fitness in the SGA screens, however, presumably reflecting redundancy in the regulation.

10.1128/mBio.01047-17.10 DATA SET S7  Changes in transcript abundance (the log_2_ fold change [FC]) and SGA fitness scores (*S*) for genes that encode transcription factors listed in the YEASTRACT database. Download DATA SET S7, XLSX file, 0.17 MB. Copyright © 2017 Hesketh et al. 2017 Hesketh et al. This content is distributed under the terms of the Creative Commons Attribution 4.0 International license.

**FIG 7:**
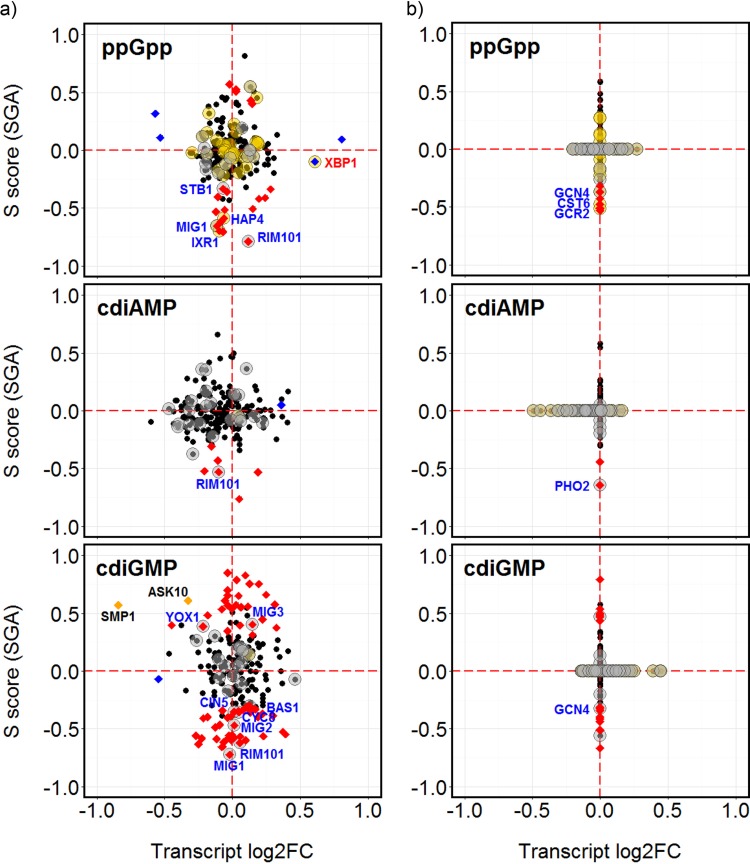
Candidate yeast transcription factors required for the adaptation to ppGpp, cdiAMP, or cdiGMP. (a) Changes in transcript abundance (the log_2_ fold change [FC]) are plotted against SGA interaction (*S*) scores for genes encoding transcription factors listed in the YEASTRACT database (39). Blue points correspond to transcripts with significantly changed abundance; red points indicate significant positive or negative interactions; orange points indicate significant changes in both transcript level and SGA interaction. Transcription factors with regulatory target sites identified as being significantly enriched in the groups of up- or downregulated genes are indicated by gold or gray halos, respectively. Names in blue text correspond to those transcription factors that exhibited both a synthetic interaction and which putatively target genes that are significantly altered in transcription. Names in red text correspond to those transcription factors that exhibited both a significant change in transcript abundance and which putatively target genes that are significantly altered in transcription. (b) A similar presentation of data for transcription factors for which only SGA fitness or microarray transcript abundance changes were available, but not both as shown in panel a. The missing data points were assigned values of 0.

Viewing the transcription factor enrichment results in the context of the SGA fitness and transcript abundance data available for the genes encoding each transcription factor provided additional support for their involvement and highlighted a possible role for Rim101p in adapting to the presence of all three nucleotides, ppGpp, cdiAMP, and cdiGMP (Fig. 7). Deletion of *RIM101*, which encodes a regulator that targets genes that are significantly altered in transcription in response to each nucleotide, also produced a synthetic sick phenotype in the presence of each nucleotide. The data similarly suggested a role for Gcn4p and Mig1p in adaptation to both of the guanine nucleotides, cdiGMP and ppGpp. All three *MIG* homologues (*MIG1* to -*3*) may be involved in adapting to the presence of cdiGMP, with deletion of *MIG1* or *MIG2* producing negative fitness interactions while loss of *MIG3* had a positive effect (Fig. 7). A *yox1* deletion also generated a positive influence on fitness in the presence of cdiGMP, while *bas1*, *cyc8*, *gis1*, or *cin5* mutations produced synthetic sick phenotypes (Fig. 7). In addition to Rim101p, SGA fitness data suggest Pho2p may also be implicated in the response to cdiAMP. Deletion of *HAP4*, which encodes a regulator involved in controlling respiratory gene expression, or *IXR1*, which controls a hypoxic response, led to reduced fitness in the presence of ppGpp. *ASK10* and *SMP1* were the only regulatory genes whose deletants exhibited significant changes in transcript abundance and caused changes in fitness; in both cases, transcription was downregulated in response to cdiGMP and the deletion mutants showed a positive interaction with cdiGMP.

### Expression of the human SpoT homologue Mesh1 prevents ppGpp accumulation in yeast and allows normal growth.

**FIG 8:**
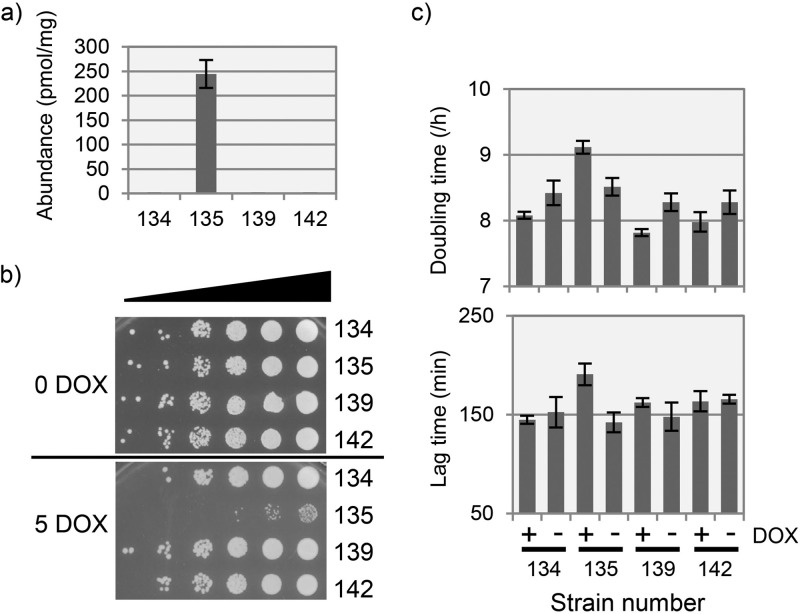
Expression of the human ppGpp hydrolase enzyme Mesh1 prevents ppGpp accumulation in yeast and allows normal growth. (a) Production of ppGpp following DOX induction in strain BY4741-112-135 (135; ppGpp synthetase) was prevented in a strain coexpressing MESH1 (BY4741-112-142; 142). Cultures were induced with DOX (5 μg/ml) and incubated for 6 h prior to analysis of intracellular nucleotide composition by LC-UV. Nucleotide abundances, normalized to picomoles per milligram (dry weight) of cells, are the averages of triplicate cultures (± the standard deviation [SD]). Strains BY4741-112-134 (134; empty vector) and BY4741-112-139 (139; Mesh1) are shown as controls. (b) Serial dilution plate growth assay of the strains shown in panel a when grown on YNB with 0.5% glucose, with (5 DOX) or without (0 DOX) 5 μg/ml DOX. Growth of strain 135 (ppGpp synthetase) was inhibited, while growth of 142 (ppGpp synthetase plus Mesh1 ppGpp hydrolase) was not. (c) DOX-induced ppGpp synthesis lengthens both doubling time and lag time in microtiter plate cultures of strain 135 (ppGpp synthetase), but coexpression of *MESH1* restored these growth characteristics to levels observed in the control strains. The data are averages (± SD) of four replicates grown in YNB with 0.5% glucose, with (+) or without (−) 5 μg/ml DOX.

Some metazoans possess a homologue of the bacterial SpoT ppGpp hydrolase enzymes that is called Mesh1, which is known to be capable of degrading ppGpp *in vitro* (40). Although deletion of Mesh1 in *Drosophila melanogaster* has been shown to retard body growth and impair stress resistance (40), metazoans are not known to synthesize ppGpp, and the biological function of Mesh1 remains unclear. To test the activity of Mesh1 toward ppGpp *in vivo*, we constitutively expressed a copy of the *Homo sapiens MESH1* coding sequence (*HDDC3*; synthesized via yeast codon usage) in *S. cerevisiae* and analyzed its effect on growth and ppGpp accumulation in strains carrying the DOX-inducible *relA* ppGpp synthetase (Fig. 8). As expected, induction of *relA* in BY4741-112-135 produced an intracellular accumulation of ca. 250 pmol of ppGpp/mg (dry weight) of cells and inhibited the growth of this strain in both solid and liquid cultures. However, the *relA*-dependent accumulation of ppGpp was reduced to zero when *MESH1* was coexpressed in strain BY4741-112-142, and growth was also restored to that observed for the control strain (BY4741-112-134). Expression of *MESH1* alone had no detectable effect on yeast intracellular nucleotide composition and showed no effect in the solid and liquid culture growth assays.

## DISCUSSION

Accumulation of the bacterial signaling nucleotides ppGpp, cdiAMP, and cdiGMP inside cells of the simple eukaryote *S. cerevisiae* inhibits cell growth (Fig. 1, 3, and 6). The observed molar ratio of production for cdiAMP:cdiGMP:ppGpp in the experiments was approximately 10:3:0.1, and thus the degree of growth inhibition they caused suggests that ppGpp and cdiGMP are the most potent molecules for disrupting eukaryotic cell physiology and cdiAMP is the least potent. The intracellular concentrations generated in this study were, with the possible exception of ppGpp, likely to be higher than could naturally be produced from an internalized bacterial population. Nevertheless, the effects on yeast cell function identified in these experiments define the spectrum of eukaryotic cellular processes which they have the potential to influence.

Both mitochondrial function and RNR activity were found to be central to the effect of cdiGMP. The chemogenomic profile for cdiGMP in the SGA screens correlated with published mitochondrial stress signatures (33), including one produced with the antiseptic trichlorophene (Data Set S2). Treatment of a human cancer cell line with trichlorophene increased generation of mitochondrial reactive oxygen species and caused a reduction in reserve oxygen capacity, confirming a mitochondrion-specific mechanism for this compound (33). Furthermore, the cytoplasmic petite mutation [rho^−^], caused by the loss of mitochondrial DNA, is synthetically lethal with cdiGMP, suggesting that either mitochondrial translation or functioning of the respiratory electron transport chain helps mitigate its toxicity in [rho^+^] strains. cdiGMP markedly increased transcription of *HUG1* and *RNR3*, two genes that can influence the activity of the RNR complex, which catalyzes the conversion of ribonucleotides to deoxyribonucleotides (38, 41). RNR plays an essential role in DNA replication and repair, and transcription of both *HUG1* and *RNR3* is known to be induced by DNA replication stress (38, 41). For all that, other hallmarks of a DNA replication stress response were not observed in the transcriptome (e.g., no upregulation of *RNR1*, *RNR2*, or *RNR4* transcription), and it may be that the effect of cdiGMP on expression of *HUG1* and *RNR3* is due to a more specific effect on inhibition of RNR activity, rather than to DNA damage. Consistent with this, inhibition of RNR by HU was synthetically lethal with cdiGMP (Fig. 6). Interestingly, there was also evidence for a regulatory association between the transcriptional repressor Mig3p and the genes downregulated in response to cdiGMP, and a *mig3* deletion produced a positive genetic interaction (Fig. 7). Derepression of Mig3p target genes is known to promote fitness in the presence of the RNR inhibitor HU (42), providing additional support for the inhibition of RNR by cdiGMP. Increased activity of RNR in yeast can upregulate the mitochondrial DNA copy number, presumably via an influence on mitochondrial deoxynucleoside triphosphate pools, suggesting this might be a rate-limiting step for mitochondrial DNA synthesis (43, 44).

Cells deleted for the *RNR1* gene are defective for respiratory growth (45). Given that growth on acetate, which is totally dependent on respiratory metabolism, is synthetically lethal with cdiGMP (Fig. 3), inhibition of RNR by cdiGMP may contribute to respiratory dysfunction. Binding sites for the related Mig1p and Mig2p transcriptional repressors, which downregulate their target genes in the presence of glucose (46), are also significantly enriched in the promoters of genes repressed by cdiGMP, but in contrast to Mig3p, both lead to the synthetic sickness phenotype when deleted (Fig. 7). Loss of Mig1p causes the derepression of genes involved in the metabolism of alternative carbon sources as well as genes involved in gluconeogenesis and respiration (47, 48), and correct repression of these processes therefore appears important for adaptation to cdiGMP. The proposed importance of Rim101p in the response to cdiGMP, and to ppGpp and cdiAMP, also implies that the nucleotides produce a disturbance in intracellular ion or pH balance, which the actions of this transcription factor helps mitigate (49).

Several lines of evidence indicate that mitochondrial function is central to coping with the presence of ppGpp: (i) deletion of genes encoding mitochondrial proteins markedly reduce cell fitness upon induction of ppGpp synthesis; (ii) ppGpp upregulates transcription of genes encoding key mitochondrial proteins, including subunits of the succinate dehydrogenase and cytochrome complexes and also Sue1p (which is required for degradation of unstable forms of cytochrome *c*); (iii) the Hap4p transcriptional activator of respiratory gene expression is implicated in both the upregulation of genes in response to ppGpp and the synthetically sick phenotype interaction that a *hap4* deletant exhibits with ppGpp biosynthesis (Fig. 7). Whether the mitochondrion contains a molecular target of ppGpp activity, or whether mitochondrial function is important for buffering an effect of the molecule exerted elsewhere, remains an open question, since the petite mutation had neither an aggravating nor alleviating effect on ppGpp activity (Fig. 3). Growth conditions necessitating exclusively respiratory metabolism similarly had no additional influence on the observed activity of ppGpp (Fig. 3). Interestingly, in plant cells, which are the only eukaryotic cells known to natively produce ppGpp, synthesis of this nucleotide is spatially separated from much of cellular metabolism, taking place in the chloroplast (50). Engineering synthesis of ppGpp in the cytoplasm of plant cells retards growth and development (51). Inhibition of yeast cell growth by ppGpp is completely reversed on heterologous expression of the human Mesh1p enzyme, which we showed to be capable of removing all the intracellular ppGpp produced (Fig. 8). The human protein atlas (http://www.proteinatlas.org) describes isoforms of Mesh1p as being expressed in all tissues, and it is interesting to speculate that it is required for abrogating the damaging effects of ppGpp introduced via bacterial infection. Consistent with this, a reduction of Mesh1 function in *Drosophila melanogaster* impairs growth and development (40). However, previous heterologous expression of a plant ppGpp synthetase in yeast demonstrated a protective effect against environmental stresses at low levels of production (10 to 20 pmol ppGpp/mg [dry weight] of cells), suggesting a concentration dependence for the effects elicited (20).

The accumulation of intracellular cdiAMP following induction of the synthetase gene exceeded the concentration observed for ATP, which is usually the most abundant nucleotide in yeast cells (Fig. 1). However, fewer and weaker genetic interactions were observed for cdiAMP synthesis than for either ppGpp or cdiGMP, and the transcriptional changes observed were no more extensive than those observed for the guanine nucleotides, which are produced at 5-fold or lower levels (Fig. 1 and 5). The bioactivity of cdiAMP therefore appears to be more limited than the bioactivities of either ppGpp or cdiGMP. Upregulation of the inorganic phosphate transporter genes *PHO84* and *PHO89*, coupled with a general enrichment for Pho2p binding sites in the promoters of the upregulated genes and the negative genetic interaction between a *pho2* deletion and cdiAMP, suggest cdiAMP induces a phosphate starvation response. This could be due to sequestration of inorganic phosphate in the high levels of cdiAMP produced. The synthetic sick genetic interactions identified for cdiAMP predominantly involved aspects of chromosome and nucleosome biology, most notably the activity of the SWR1 complex (Fig. 1; Data Set S3). Petite mutations and growth on acetate also produced synthetic sickness (Fig. 3), but no interaction was observed with the RNR inhibitor HU (Fig. 6). The SWR1 complex replaces histone H2A in nucleosomes with the H2AZ variant which influences a range of processes, including transcription, DNA repair, and chromosome segregation (52). Abrogation of SWR1 complex function was detrimental to cell fitness in the presence of all three nucleotides, possibly attributable to the role of the complex in the induction of the expression of stress-responsive genes (53). In contrast, histone H3K4 methylation inhibits transcription of stress-responsive genes (53), and deletion of genes required for this process produced positive genetic interactions for cdiGMP (Data Set S5). Loss of the SWI/SNF chromatin remodeling complex activity and deletion of *SGF73*, each known to produce a reduction in respiratory activity, had positive effects on fitness in all cases (Fig. 4).

Another group of genes that produce positive interactions with more than one nucleotide are a subset of those encoding ribosomal proteins (Fig. 3). In yeast, the majority of genes encoding cytosolic ribosomal proteins are duplicated. There is extensive variation in both expression and function of the paralogues, and the existence of “specialized” ribosomes has been proposed, with different subsets of duplicated ribosomal proteins producing ribosomes with subtly different functions (54, 55). Deletion of paralogues yielding positive interactions (*RPL11B*, *RPL14A*, *RPL22A*, *RPL23A*, and *RPS0B*) suggests that the generation of ribosome types with an increased proportion of the alternative version of the protein serves to increase fitness in the presence of the nucleotides. Another possible explanation would be the existence of extraribosomal “moonlighting” functions for these paralogues (56), although none has been reported thus far.

### Concluding remarks.

cdiGMP, cdiAMP, and ppGpp are each capable of adversely affecting yeast cell function and growth. In addition to the induction of host immune responses, the presence of these molecules inside infected cells of higher eukaryotes therefore has the potential to mediate damaging effects. The molecular basis for the complex effects produced on yeast cell function are yet to be clearly defined, but mitochondrial function and chromatin remodeling and modification processes serve as important buffers against their activity. Respiratory competence and RNR activity are particularly important with respect to cdiGMP. The clearance of ppGpp from yeast cells by the human Mesh1p ppGpp hydrolase enzyme restores wild-type growth and suggests that higher eukaryotes may have evolved systems for eliminating at least one of the damaging bacterial nucleotides from their cells.

## MATERIALS AND METHODS

### Plasmid constructs and yeast strains.

Plasmids and strains used in this study are listed in Table 2. To produce plasmids for DOX-inducible bacterial nucleotide synthesis in yeast, the following DNA sequences were cloned into plasmid pCM252 downstream of the TetO7 promoter present in the vector (25). For ppGpp synthesis, a 1.4-kb N-terminal sequence of the *relA* gene (28) was amplified from genomic DNA of *E. coli* JM101 by using the primers 5′-CACACACTAAATTACCGGATCAATTCGGGGGATCCATGGTTGCGGTAAGAAGTGCA-3′ and 5′-TACATGATGCGGCCCTCCTGCAGGGCCCTATTACAGCTGGTAGGTGAACGGCAC-3′. This sequence was cloned by yeast gap repair cloning into pCM252 (25), previously linearized by digestion with HpaI and NotI, generating pAH12. For cdiAMP synthesis, coding sequence from the *ybbP* gene (27) was amplified from genomic DNA of *B. subtilis* JH642 by using the primers 5′-GATCGGATTCATGGCTTTTGAGGATATCCCT-3′ and 5′-GATCGTTAACTTATCCATTTTTCCTGCCCCT-3′ and then digested and cloned between the BamHI-HpaI sites of pCM252, producing pAH36. A synthetic gene designed to encode the cdiGMP synthetase variant DgcA0244 from *C. crescentus* (26) via yeast codon usage and possessing a Kozak consensus sequence immediately upstream of the ATG start codon was similarly cloned into pCM252 between the HpaI-NotI sites, producing pAH71 for cdiGMP synthesis. pAH12, pAH36, and pAH71 were transformed into CML282 containing an integrated copy of the SSN6::TetR fusion construct pCM242 (25, 29). For experiments in the BY4741 genetic background, the TetO7pr::*relA*, TetO7pr::*ybbP*, and TetO7pr::*dgcA* genes (with their VP16::TetR′ TetO7 transactivator sequences) were cloned separately into a centromeric vector, pRS-*LEU2*-*URA3* that is doubly marked with the yeast *LEU2* and *URA3* genes to generate pAH135, pAH136, and pAH137, respectively (Fig. S3). Similar cloning of the empty TetO7pr::sequence from pCM252 generated the control plasmid pAH134. pRS-LEU2-URA3 was created by cloning the *URA3* gene amplified from pRS416 (57) by using the primers 5′-GACAGGCGCCGATTCGGTAATCTCCGAACAGA-3′ and 5′-GACTGCCGGCTGATGCGGTATTTTCTCCTTA-3′ between the SfoI and NgoMIV sites of pRS415 (57). For ppGpp hydrolysis, a synthetic coding sequence designed to specify the Mesh1 enzyme from *Homo sapiens* (40) with yeast codon usage was cloned into a vector between the promoter and terminator sequences of the yeast gene *TDH3*. TetO7pr::*relA* (with the VP16::TetR′ TetO7 transactivator sequence) and *TDH3*pr::MESH1 expression constructs were then combined in pRS-*LEU2*-*URA3* to produce pAH142, and also the control plasmid pAH139, carrying only the *mesh1* gene construct (Fig. S3). This series of centromeric plasmids were transformed into yeast strain BY4741 or Y7092 (24), each carrying an integrated copy of the SSN6::TetR fusion repressor construct from pCM242 (25) on plasmid pAH112. The integrative plasmid pAH112 was constructed by cloning the *MET15* gene amplified from pRS411 (58) using the primers 5′-GACACCCGGGATGCGCCATCCTCATGAAAACTGTGT-3′ and 5′-GACTGGATTCCTTGTGAGAGAAAGTAGGTTTATACA-3′ between the XmaI and BamHI sites of pRS303 (57), then adding the required 4,162-bp XhoI-SphI DNA fragment from pCM242 (25).

10.1128/mBio.01047-17.3 FIG S3  Summary of the centromeric plasmids generated in this study for the synthesis and degradation of bacterial nucleotides in *S. cerevisiae*. Download FIG S3, PDF file, 0.1 MB. Copyright © 2017 Hesketh et al. 2017 Hesketh et al. This content is distributed under the terms of the Creative Commons Attribution 4.0 International license.

**TABLE 2:** Plasmids and yeast strains used in this study

Plasmid or strain	Description or relevant genotype	Reference
Plasmids		
pCM252	TRP1 VP16::TetR' TetO7pr	25
pAH12	pCM252 TetO7pr::*relA*	This work
pAH36	pCM252 TetO7pr::*ybbP*	This work
pAH71	pCM252 TetO7pr::*dgcA*	This work
pAH112	pRS-HIS3-MET15 TetR::SSN6	This work
pAH134	pRS-LEU2-URA3 VP16::TetR' TetO7pr	This work
pAH135	pRS-LEU2-URA3 VP16::TetR' TetO7pr::*relA*	This work
pAH136	pRS-LEU2-URA3 VP16::TetR' TetO7pr::*ybbP*	This work
pAH137	pRS-LEU2-URA3 VP16::TetR' TetO7pr::*dgcA*	This work
pAH139	pRS-LEU2-URA3 VP16::TetR' TetO7pr::TDH3pr::*MESH1*	This work
pAH142	pRS-LEU2-URA3 VP16::TetR' TetO7pr::*relA* TDH3pr::*MESH1*	This work
Strains		
CML282	BMA64-1A *MAT***a** *leu2-3 ade2-1 trp1-1 ura3-1 can1-100 his3-11,15 LEU2*::(TetR::SSN6)	25
CML282-252	CML282 with plasmid pCM252	
CML282-12	CML282 with plasmid pAH12	This work
CML282-36	CML282 with plasmid pAH36	This work
CML282-71	CML282 with plasmid pAH71	This work
BY4741	*MAT***a** *ura3Δ0 leu2Δ0 met15Δ0 his3Δ1*	
BY4741-112	BY4741 with integrated plasmid pAH112 (*his3Δ1*::pAH112 *ura3Δ0 leu2Δ0*)	
BY4741-112-134	BY4741-112 with centromeric plasmid pAH134 (prototroph)	This work
BY4741-112-135	BY4741-112 with centromeric plasmid pAH135 (prototroph)	This work
BY4741-112-136	BY4741-112 with centromeric plasmid pAH136 (prototroph)	This work
BY4741-112-137	BY4741-112 with centromeric plasmid pAH137 (prototroph)	This work
BY4741-112-139	BY4741-112 with centromeric plasmid pAH139 (prototroph)	This work
BY4741-112-142	BY4741-112 with centromeric plasmid pAH142 (prototroph)	This work
Y7092	*MAT***a** *his3*Δ*1 leu2*Δ*0 ura3*Δ*0 met15*Δ*0 can1*Δ::*STE2pr-Sp_his5 lyp1*Δ	24
Y7092-112	Y7092 with integrated plasmid pAH112 [*his3*Δ*1*::pAH112(*his3*Δ) *ura3*Δ*0 leu2*Δ*0*]	This work
Y7092-112-134	Y7092-112 with centromeric plasmid pAH134 [*his3*Δ*1*::pAH112(*his3*Δ)]	This work
Y7092-112-135	Y7092-112 with centromeric plasmid pAH135 [*his3*Δ*1*::pAH112(*his3*Δ)]	This work
Y7092-112-136	Y7092-112 with centromeric plasmid pAH136 [*his3*Δ*1*::pAH112(*his3*Δ)]	This work
Y7092-112-137	Y7092-112 with centromeric plasmid pAH137 [*his3*Δ*1*::pAH112(*his3*Δ)]	This work
BY4741 mutant collection	*MAT***a** *ura3Δ0 leu2Δ0 met15Δ0 his3Δ1*::KANMX4	69

### Analyzing the activity of engineered strains based on DOX induction in batch culture.

Flasks (250 ml) of YNB minimal medium (0.67% yeast nitrogen base [Sigma] and 0.5% [wt/vol] ammonium sulfate) containing 0.5% glucose and DOX (5 or 10 μg/ml, as indicated) were inoculated to a starting optical density at 600 nm (OD_600_) of 0.05 from overnight cultures. When required, a similar set of flasks lacking DOX was also inoculated for comparison as noninduced controls. The inoculated cultures were incubated at 30°C with shaking at 200 rpm until they reached an OD_600_ of 0.4 to 0.6 and then sampled for intracellular nucleotide extraction and, when required, RNA. For growth assays in microtiter plate cultures, inoculated medium was dispensed into a 96-well plate, and cell density was monitored in triplicate as the OD_595_ by using a plate reader incubator (BMG Biotech).

### Analysis of intracellular nucleotides.

Intracellular nucleotide extracts were prepared and analyzed by high-performance liquid chromatography with a UV detector (HPLC-UV) or LC-mass spectrometry (MS). Briefly, cell metabolism in culture samples was immediately quenched by transferring culture aliquots directly to methanol (>3× sample volume) that had been cooled to below −60°C. Quenched cells were extracted on ice using 1 N formic acid containing 10% (vol/vol) butan-1-ol, and then the extracts were filtered, frozen, and freeze-dried. Nucleotides in the reconstituted extracts were separated and quantified using gradient anion-exchange chromatography and either UV diode array detection at 254 nm (59) or detection by mass spectrometry using an Agilent 6460 triple-quad mass spectrometer in multiple reaction monitoring (MRM) mode (60). Quantification was achieved by comparison to known standards, normalizing values (in picomoles) to the dry weight of cells present in 1 ml of a culture with an OD_600_ of 1. ATP and GTP nucleotide standards were purchased from Sigma, cdiAMP and cdiGMP were obtained from Biolog, and ppGpp was from Trilink Biosciences. For the LC-MS analysis, MRM mass transitions (reported as ratio of the parent ion mass to fragment ion mass) used for detection and identification of the nucleotides in the positive ion mode were as follows: ppGpp, 604.1 to 152.1; cdiAMP, 659.4 to 136.1; cdiGMP, 691.4 to 152.2; ATP, 508.1 to 136.0; GTP, 524.2 to 152.0.

### Construction and screening of SGA libraries.

Synthetic genetic arrays were generated to interrogate the activity of the pAH135 (ppGpp synthesis), pAH136 (cdiAMP synthesis), and pAH137 (cdiGMP synthesis) plasmid vectors according to the methodology of Tong and Boone (24) as outlined below. Plasmid pAH134 was included as a negative control. For each array, query strains were constructed by first integrating pAH112 carrying the TetR::SSN6 TetO7 repressor (25) into the *his3*Δ*1* locus of Y7092 (24) to generate the base query strain Y7092-112. For transformation, pAH112 was linearized within its *HIS3* gene by using BsiWI to ensure that integration did not restore a functional copy of *HIS3* and that Y7092-112 remained auxotrophic for histidine (to enable future retrieval of *MAT***a** haploids from the SGA matings via the activity of the STE2pr-Sp_*his5* construct present in Y7092). Transformation of Y7092-112 with each centromeric vector of interest generated the final *MATα* query strains (Table 1). Query strains were mated with the BY4741 *MAT***a** nonessential mutant library (each with an open reading frame replaced by a KANMX4 antibiotic resistance cassette) arrayed in a 384 format (24 columns by 16 rows; 5,362 arrayed strains representing 4,978 different mutants) by robotic replica pinning (Singer Rotor HDA robot). Diploids were selected on 2% glucose YNB (0.67% [wt/vol] yeast nitrogen base [Sigma]) minimal Met Leu Ura dropout medium containing 0.1% (wt/vol) monosodium glutamate as nitrogen source and the antibiotic G418 (400 μg/ml) for *Kan*MX4 marker selection. After sporulation, *MAT***a** progeny were isolated by selection on 2% glucose YNB (0.67% [wt/vol] and 0.5% [wt/vol] ammonium sulfate) minimal Arg Lys His dropout medium containing canavanine (50 μg//ml) and thialysine (50 μg/ml). Final rounds of selection on 2% glucose YNB (0.67% [wt/vol]) minimal His Met Leu Ura dropout medium containing 0.1% (wt/vol) monosodium glutamate and G418 (400 μg/ml) yielded the desired output arrays of strains containing the mutated test gene, the integrated pAH112 construct, and the centromeric query vector.

Screens were performed by pinning the arrays in quadruplicate (final 1,536 format) to replica plates of YNB (0.67% [wt/vol] and 0.5% [wt/vol] ammonium sulfate) minimal medium containing 0.5% glucose with or without DOX (5 μg/ml). Plates were incubated at 30°C for 3 days before being scanned. Scanned images were then processed and analyzed to measure any changes in colony size between the DOX-induced and the noninduced plates.

### SGA screen data analysis.

Genetic interactions between null mutations in nonessential yeast genes and induction of ppGpp, cdiAMP, or cdiGMP synthesis were identified by comparing colony sizes (as a proxy for fitness) between the DOX-induced and the noninduced plates for query plasmids pAH135, pAH136, and pAH137, respectively, relative to the control query using plasmid pAH134. Colony areas on scanned images were quantified, spatially normalized, row-column normalized, and then filtered using the R package gitter (61) and the R script SGAtools (62). Plate normalization was not used, as it makes the assumption that changes in colony size are rare events (62), which is incorrect for these SGA induction screens (Fig. 1b and c). Jackknife filtering flagged colonies contributing more than 90% of the variance in their four replicate spots for removal, and a low-area filter was used to finally remove any set of mutant replicate spots with a median area below a minimum threshold in any of the SGA screens (sum of the median areas on the DOX and no-DOX plates of <400).

Multiplicative interaction scores, defined by the equation *S* = *Wij* – *Wi* × *Wj* (62) were calculated for the normalized filtered data (replicates for 4,990 arrayed strains corresponding to 4,639 different mutants) in R, based on the following definitions: the observed interaction effect between a mutation in gene *x* and DOX induction of expression from each query plasmid *q*, *Wij*, is given by the ratio of the median area of mutant *x* colonies on the plate containing DOX versus the median on the no-DOX control plate for the query strain *q*; the expected effect of DOX induction of expression from each query plasmid *q*, *Wi*, is given by the ratio of the median area of all mutant colonies on the DOX plates versus the median of all on the no-DOX control plates for the query strain *q*; and the expected effect of gene mutation for each mutant, *Wj*, is given by the ratio of the median area of all mutant *x* colonies on the no-DOX plates for all query strains to the median area of all mutant colonies on the no-DOX plates for all query strains. In addition to the *S* scores, evidence for interaction effects between a mutation in gene *x* and DOX induction of expression from each query plasmid was sought by testing the colony areas for significant differences using the Treat function within Limma (63), assuming a null hypothesis where the size differential for mutant *x* colonies between the DOX-induced and no-DOX control plates was the same as the median of the size differentials observed for all mutants. The Limma Treat function analysis threshold was set at 1.3 to test for mutants showing significant differences greater than 1.3-fold from the expected values. Mutations that positively or negatively interacted with the synthesis of a particular nucleotide were finally defined via comparisong with the mutants showing no interaction in the control query strain pAH134 (0.7 ≥ *Wij* of pAH134 ≤ 1.3; 4,916 arrayed strains corresponding to 4,574 different mutants) as those with a Limma analysis *q*-value of ≤0.05 and *S* scores of ≤0.3 (positive interaction) or less than or equal to −0.3 (negative interaction). The log_2_ fold change values for SGA interactions determined for each strain were used to calculate the FD scores for correlation with published FD scores derived from chemogenomic profiling analysis of homozygous diploid mutants (33). Significant Pearson correlations (*q* < 0.01) were obtained by adjusting correlation *P* values using the Benjamini and Hochberg correction.

### Isolation of petite mutants.

Petite mutants were isolated from strains plated onto YNB (0.67% [wt/vol] and 0.5% [wt/vol] ammonium sulfate) minimal medium containing 2% glucose and 5 μg/ml ethidium bromide. Independently isolated mutants were verified as [*rho*0] by assaying for the inability to grow on a nonfermentable carbon source (0.5% acetate) and by the failure to PCR amplify the mitochondrial DNA gene *COX1* from a total DNA extract, relative to an *ACT1* genomic DNA control.

### Interaction analysis of petite strains and of the effect of hydroxyurea.

Screens of the petite strains were performed by robotically pinning four verified independent isolates in octuplicate (final 384 format) to replica plates of YNB (0.67% [wt/vol] and 0.5% [wt/vol] ammonium sulfate) minimal medium containing 0.5% glucose or 2% glucose, with and without DOX (5 μg/ml). Plates were incubated at 30°C for 4 days before being scanned. The parental *rho*^+ ^strains were analyzed under the same conditions as the reference strains but were grown for 3 days before scanning and were pinned as 24 replicates (final 384 format). The latter also formed part of the screen for the effect of HU, which included pinning to plates with 100 mM HU plus 0 μg/ml DOX or 100 mM HU plus 5 μg/ml DOX.

Scanned images were then processed and analyzed to measure any changes in colony size. Colony areas on scanned images were quantified and spatially normalized using gitter and SGAtools, as described above. Multiplicative interaction scores defined by the equation *S* = *Wij* – (*Wi* × *Wj*) (62) were calculated to identify whether petiteness or HU treatment synthetically alleviated (positive interaction) or aggravated (negative interaction) the expected fitness. For the petite strains, *Wi* represents the effect of the petite mutation on colony area relative to the parental rho^+^ strains; *Wj* is the effect of DOX induction on colony area in the rho^+^ strains; and *Wij* is the effect of DOX induction on colony area in the petite [rho^−^] strains. For the HU treatment, *Wi* represents the effect of HU on colony area relative to untreated controls; *Wj* is the effect of DOX induction on colony area; and *Wij* is the effect of DOX induction in the presence of HU on colony area. *S* scores around 0 indicate no synergy, while positive or negative values indicate the degree of positive or negative interaction, respectively.

### Transcriptomics analysis.

Strains BY4741-112-134, BY4741-112-135, BY4741-112-136, and BY4741-112-137 were grown in triplicate in induced (5 μg/ml DOX) or noninduced (0 μg/ml DOX) batch cultures and sampled at mid-exponential phase (OD_600_ of 0.4 to 0.6) for RNA and intracellular nucleotide extraction. For RNA isolation, cells from culture samples were harvested rapidly by centrifugation, and the cell pellets were flash-frozen in liquid nitrogen and stored at −80°C until required. For RNA purification, frozen cells were resuspended in Trizol (Invitrogen) and lysed mechanically at 4°C by bead beating using a FastPrep homogenizer (MP Biomedicals; five 1-min cycles of shaking at 6 m/s). DNA and protein were removed from the lysed samples by extraction with chloroform (twice), and total RNA was purified by using RNeasy columns (Qiagen). Total RNA was processed for hybridization to Affymetrix Yeast 2.0 microarrays using the Affymetrix 3′ IVT Express kit according to the manufacturer’s instructions. *S. cerevisiae* probe sets on scanned microarray data files were normalized based on the robust multiarray average (RMA) (64), filtered to remove probes exhibiting low signals or low variance across the experiment, and then tested for differential expression using the Treat function with Limma, with a fold change threshold (up- or downregulation) of ≥10% (63). Filtering kept only those genes with expression values above twice the average background in at least a quarter of the arrays (removing 410/5,900) and then selected the 75% most variable genes.

### Other computational methods.

Networks were constructed in Cytoscape v3.3 (65), and analysis of regulation by transcription factors was performed using the YEASTRACT database (39). Analyses of correlations between data sets and of gene ontology enrichment were performed in R (66) using the base package and the GOstats Bioconductor package (67). Analysis of growth curves obtained from the plate reader experiments was performed in R using the grofit package (68).

### Accession number(s).

Microarray data from this study are available in the ArrayExpress database (http://www.ebi.ac.uk/arrayexpress) under accession number E-MTAB-5174.
