# Supplementary material for: Bacterial Signaling Nucleotides Inhibit Yeast Cell Growth by Impacting Mitochondrial and Other Specifically Eukaryotic Functions
Source: mBio. 2017 Jul 25;8(4):e01047-17. doi: 10.1128/mBio.01047-17 (PMC5527313; doi:10.1128/mBio.01047-17)
Supplement: FIG S1 [file mbo004173404sf1.pdf]

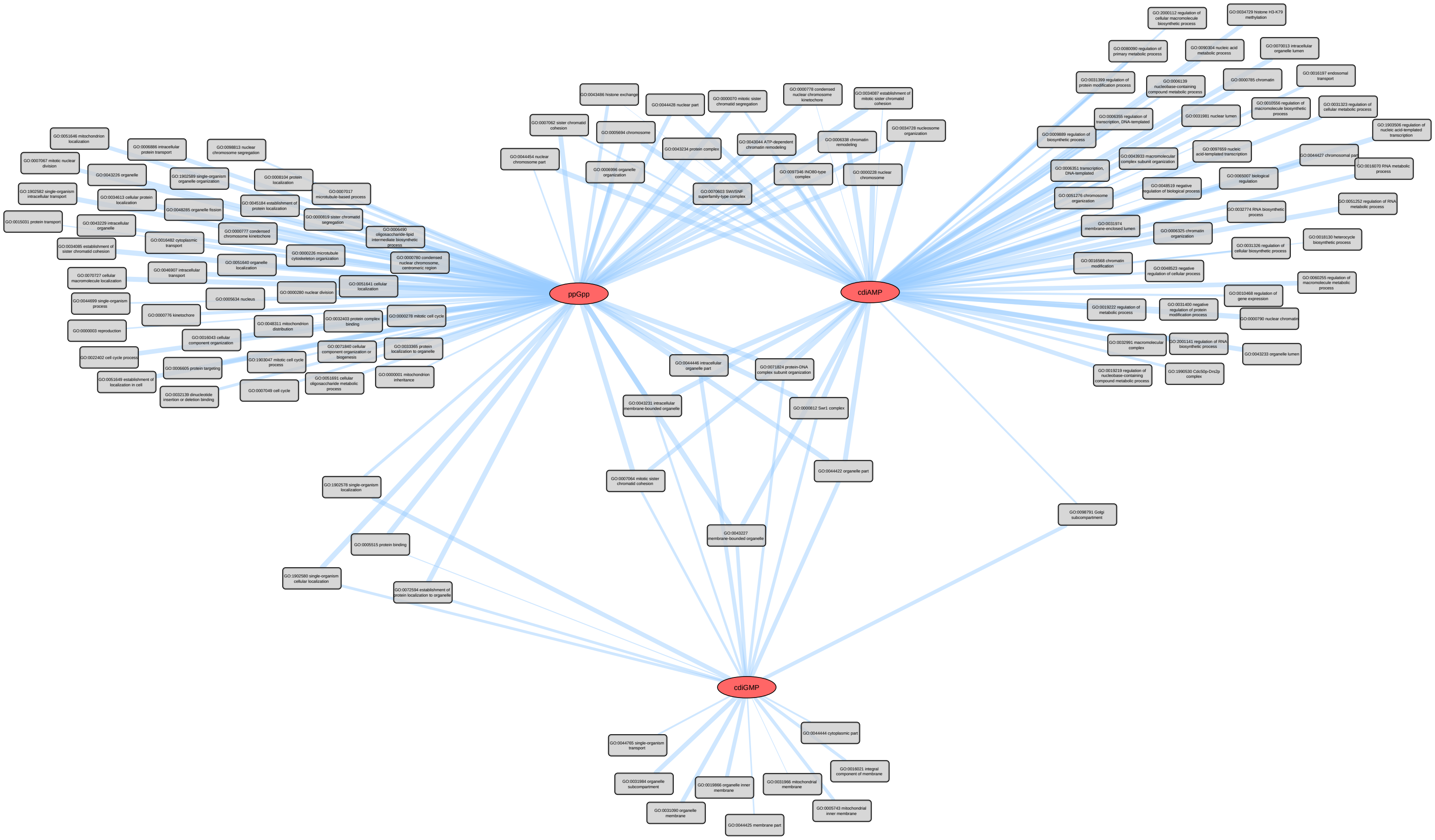

Supplementary Fig. S1. Network of GO categories significantly (P<=0.001) enriched in the groups of genes showing synthetic negative interactions with each nucleotide (based on GO analysis results presented in Supplementary file 1). Line thickness is proportional to P-value, with thicker lines being more significant.
