# Supplementary material for: Bacterial Signaling Nucleotides Inhibit Yeast Cell Growth by Impacting Mitochondrial and Other Specifically Eukaryotic Functions
Source: mBio. 2017 Jul 25;8(4):e01047-17. doi: 10.1128/mBio.01047-17 (PMC5527313; doi:10.1128/mBio.01047-17)
Supplement: FIG S2 [file mbo004173404sf2.pdf]

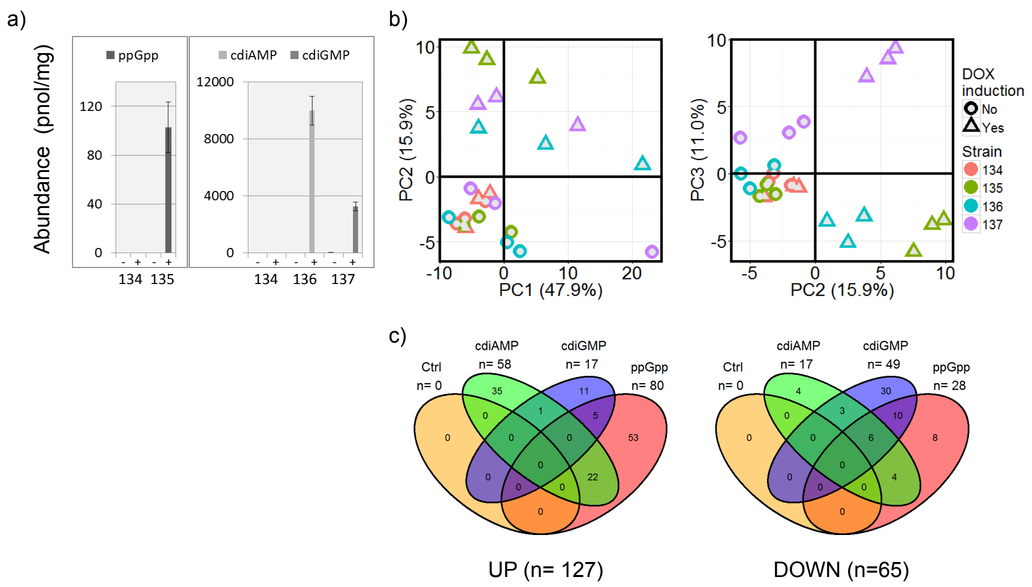

Changes in yeast gene expression caused by the bacterial nucleotides ppGpp, cdiAMP and cdiGMP. a) Liquid cultures treated (+) or not treated (-) with 5 µg/ml DOX were grown to mid-exponential phase, and analysed for bacterial nucleotide synthesis by HPLC-UV. b and c) RNA was isolated from aliquots of the cultures, and mRNA abundances quantified using Affymetrix microarrays. PCA analysis (b) indicates changes in the pattern of gene transcription in the presence of each bacterial nucleotide which were identified by differential expression testing using limma treat (q<=0.05, 10% fold change threshold). Venn diagrams (c) summarise the genes identified as being significantly up or down regulated by each nucleotide.

Supplementary Fig. S2
