# Supplementary material for: Bacterial Signaling Nucleotides Inhibit Yeast Cell Growth by Impacting Mitochondrial and Other Specifically Eukaryotic Functions
Source: mBio. 2017 Jul 25;8(4):e01047-17. doi: 10.1128/mBio.01047-17 (PMC5527313; doi:10.1128/mBio.01047-17)
Supplement: FIG S3 [file mbo004173404sf3.pdf]

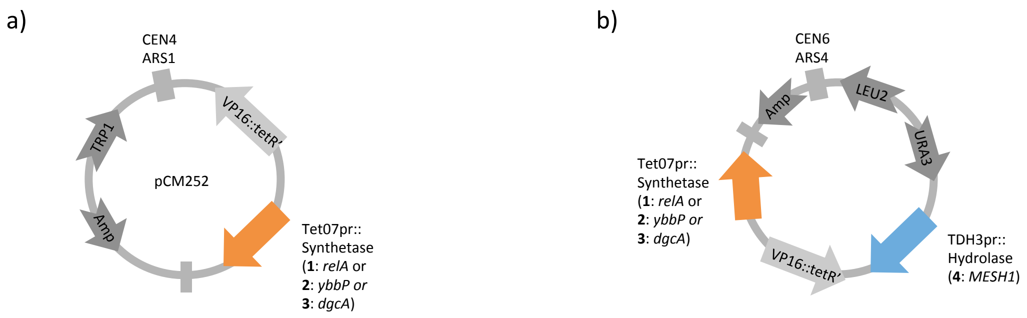

**Reaction**	**Enzyme**	**Encoded in plasmid/strain number**						
		a)	252	12	36	71		
		b)	134	135	136	137	139	142
1: ATP+GTP → (p)ppGpp	*Escherichia coli* RelA			✓				✓
2: ATP+ATP → cdiAMP	*Bacillus subtilis* YbbP				✓			
3: GTP+GTP → cdiGMP	*Caulobacter crecentus* DgcA					✓		
4: ppGpp → GDP+ppi	*Homo sapiens* Mesh1p						✓	✓

Summary of the centromeric plasmids generated in this study for the synthesis and degradation of bacterial nucleotides in *S. cerevisiae*.

Supplementary Fig. S3
